# Calcium:Magnesium Ratio in Local Groundwater and Incidence of Acute Myocardial Infarction among Males in Rural Finland

**DOI:** 10.1289/ehp.8438

**Published:** 2006-01-13

**Authors:** Anne Kousa, Aki S. Havulinna, Elena Moltchanova, Olli Taskinen, Maria Nikkarinen, Johan Eriksson, Marjatta Karvonen

**Affiliations:** 1 Geological Survey of Finland, Kuopio, Finland; 2 Department of Epidemiology and Health Promotion, National Public Health Institute, Helsinki, Finland

**Keywords:** acute myocardial infarction, Bayes, Ca, Ca:Mg ratio, Cr, Mg, small-area study

## Abstract

Several epidemiologic studies have shown an association between calcium and magnesium and coronary heart disease mortality and morbidity. In this small-area study, we examined the relationship between acute myocardial infarction (AMI) risk and content of Ca, Mg, and chromium in local groundwater in Finnish rural areas using Bayesian modeling and geospatial data aggregated into 10 km × 10 km grid cells. Data on 14,495 men 35–74 years of age with their first AMI in the years 1983, 1988, or 1993 were pooled. Geochemical data consisted of 4,300 measurements of each element in local groundwater. The median concentrations of Mg, Ca, and Cr and the Ca:Mg ratio in well water were 2.61 mg/L, 12.23 mg/L, 0.27 μg/L, and 5.39, respectively. Each 1 mg/L increment in Mg level decreased the AMI risk by 4.9%, whereas a one unit increment in the Ca:Mg ratio increased the risk by 3.1%. Ca and Cr did not show any statistically significant effect on the incidence and spatial variation of AMI. Results of this study with specific Bayesian statistical analysis support earlier findings of a protective role of Mg and low Ca:Mg ratio against coronary heart disease but do not support the earlier hypothesis of a protective role of Ca.

Since the 1950s, several epidemiologic studies have demonstrated an inverse relation between water hardness and cardiovascular disease (CVD) (Crawford et al. 1968; Kousa and Nikkarinen 1997; Kousa et al. 2004; Masironi et al. 1980; Nerbrand et al. 1992; Piispanen 1993), whereas some other studies have not found such a relationship (Miyake and Iki 2004; Rosenlund et al. 2005). Drinking-water content of magnesium and calcium has been shown to reduce the risk of CVD (Luoma et al. 1983; MacPherson and Bacsó 2000; Marque et al. 2003; Punsar and Karvonen 1979; Rubenowitz et al. 1996, 2000; Rylander et al. 1991). The cardiovascular conditions associated with Mg deficiency include myocardial infarction, hypertension, congestive heart failure, and arrhythmias (Altura and Altura 1991–92; Gums 2004; Saris et al. 2000).

In Finland, the within-country variation of the coronary heart disease (CHD) mortality and morbidity is well established (Karvonen et al. 2002; Näyhä 1989). In the 1980s, CHD risk was 40% higher in eastern Finland than in the western parts of the country (Jousilahti et al. 1998). The availability of some cardioprotective substances, such as Ca, Mg, and chromium in soil and dissolved in water, has been suggested to be associated with the geographic variation of CHD in Finland (Karppanen and Neuvonen 1973; Karppanen et al. 1978; Punsar et al. 1975). Also, Ca:Mg ratio in the diet has been suggested to be related to CHD mortality (Karppanen et al. 1978). The decline of the major CHD risk factors since the mid-1960s has been accompanied by decline in CHD mortality and morbidity (Pyörälä et al. 1985; Salomaa et al. 1996; Vartiainen et al. 1994). A recent study of the spatial distribution of the first acute myocardial infarction (AMI) event showed that despite the decreasing trend in AMI incidence, the geographic difference in incidence and high-risk areas still exist in Finland (Karvonen et al. 2002). The major risk factors of CHD, such as serum cholesterol, blood pressure, and smoking, do not adequately explain the geographic variation of CHD risk in Finland (Jousilahti et al. 1998; Vartiainen et al. 2000). One possible explanation of regional variation could be some environmental risk factors that have accumulated in certain areas in Finland.

Several properties or compounds in groundwater reflect the mineral composition of the bedrock or the soil it is derived from (Lahermo et al. 1990). The hydrogeochemical surveys carried out in Finland have shown that geographic differences in the levels of geochemical compounds are very stable and did not smooth during long periods (Korkka-Niemi et al. 1993; Lahermo et al. 1990; Tarvainen et al. 2001). The regional distribution of Ca and Mg in groundwater is quite similar to total water hardness. Regionally, the hardest groundwater has been reported in the southern Finland coastal belt, whereas the softest waters are in northeast Finland and Lapland (Tarvainen et al. 2001). The median Cr content in Finnish bedrock varies from 2,300 mg/kg in mafic rocks to 4 mg/kg in granitic rock (Koljonen 1992). Cr is naturally mainly bound in resistant minerals, and the median content in Finnish soil is 60 mg/kg. Cr is present in the environment in several different forms. The most common form in soil is Cr^3+^, and its compounds are very stable in the circumstances that prevail in Finnish soils (Kabata-Pendias and Pendias 2001). As a consequence, the Cr concentrations in well waters are generally low, with a mean content of 0.3 μg/L (Tarvainen et al. 2001).

In a previous study (Kousa et al. 2004), we showed an inverse association between total water hardness and geographic variation of the AMI incidence in Finland. In the present study, our aim was to examine Ca and Mg, the main elements contributing to water hardness, in local groundwater and their associations with the geographic variation of AMI incidence among men 35–74 years of age in rural areas in Finland. Furthermore, we examined the association of Cr in local ground-water and the geographic variation of AMI.

## Materials and Methods

A total of 14,495 men 35–74 years of age living in rural areas of Finland participated in the study. Information on their first AMI was obtained from the National Death Register and the Hospital Discharge Register (National Research and Development Centre for Welfare and Health, Helsinki, Finland). Every Finnish citizen has a unique personal identification number. This national personal identification number was used to perform a computerized record linkage of the data for deaths and hospitalization due to AMI (codes 410–414 of the *International Classification of Diseases*, revisions 8 and 9) [World Health Organization (WHO) 1965, 1975]. Both fatal and nonfatal events from 1983, 1988, and 1993 were included. The records were linked to trace the possible earlier events of AMI in each case obtained from the National Death Register and the Hospital Discharge Register. Cases with a previous hospitalization for AMI were excluded. In addition, employing identification number, each citizen of rural Finland was localized according to the map coordinates of the place of residence at the time of diagnosis. The data on population at risk, provided by coordinates of the place of residence, were obtained from Statistics Finland (Helsinki, Finland).

The urban–rural status of an area was defined employing dichotomous classification, using the following guidelines. Area was considered urban if it contained only few or no sparsely populated areas, or if its population in built-up areas exceeded 15,000 inhabitants. The rest of the study area was considered rural (Keränen et al. 2000).

Data on Ca, Mg, and Cr were obtained from the hydrogeochemical database of the Geological Survey of Finland (Lahermo et al. 1990). Ca and Mg were determined using the inductively coupled plasma atomic emission spectrometry method, and Cr concentrations with the ICP-mass spectrometry method. Ca, Mg, and Cr measurements (*n* = 4,300) were interpolated into a regular 10 × 10 km grid over Finland using a hierarchical Bayesian conditional autoregressive (CAR) model (Appendix 1), which allowed us to consider the uncertainty of each observation. The posterior means in each grid cell were taken as the results of the interpolations.

A full Bayesian CAR model with covariates was applied for cross-section years 1983, 1988, and 1993 (Appendix 2). A contiguous neighborhood structure, 10 × 10 km grid cells excluding Lapland, Ahvenanmaa, and Turku Archipelago, was given for the CAR prior. The neighbors were defined to be all cells adjacent to cell *i* by side or corner. Only nonurban grid cells were included when calculating the Poisson likelihood because people in the non-urban areas mostly use well water, whereas the urban dwellers use public tap water.

In this analysis, age was divided into eight 5-year age groups: 35–39, 40–44, 45–49, 50–54, 55–59, 60–64, 65–69, and 70–74. A nonproportional hazard model described the age group effect, which for AMI is more appropriate than the proportional hazards (Karvonen et al. 2002). The posterior distributions of the parameters of interest were estimated and summarized. To describe the variability of the estimates, we used a 95% highest density region (HDR), which is defined as the most compact set of parameter values supporting 0.95 of the posterior mass and is a Bayesian counterpart to a classical (frequentist) confidence interval.

The models were fitted using WinBUGS (Spiegelhalter et al. 2004). We ran a total of 10,000 iterations with 5,000 burn-ins. “Burn-in” denotes iterations that were discarded due to nonconvergence of the model at the early stages of the algorithm. The convergence was assessed visually from the sample paths.

## Results

Age group; Ca, Mg, and Cr concentrations; and Ca:Mg ratio in groundwater (well water) were included in the spatial models as covariates. The overall age-standardized incidence of AMI among men 35–74 years of age in nonurban area was 503/100,000/year (posterior 95% HDR, 494–511). In the interpolated grid data, Mg and Ca varied in local groundwater from 1.00 to 9.77 mg/L and 4.39 to 37.37 mg/L, respectively. The Ca:Mg ratio varied from 2.16 to 11.66. The correlation of Ca and Mg was very strong (*r* = 0.85). Interpolated Cr concentrations varied from 0.13 to 1.10 μg/L. All Cr concentrations were < 50 μg/L, which is the recommended guideline of drinking water for Cr (WHO 1993). Basic descriptive statistics of geochemical constituents of groundwater are provided in Table 1. Tables 2 and 3 illustrate the number of AMI cases, population at risk, and the AMI incidence by age and interpolated geochemical covariates of the local groundwater. The age-standardized AMI incidence was highest (622/100,000/year; posterior 95% HDR, 591–649) in the lowest tertile (Mg < 2.28 mg/L) of well water Mg content compared with the risk with higher Mg levels (Table 2). In the highest tertile of Ca:Mg ratio (Ca:Mg ratio > 5.73), the age-standardized AMI incidence was highest (586/100,000/year; posterior 95% HDR, 564–606) compared with the risk with lower Ca:Mg ratios (Table 3). The relations of Mg level and Ca:Mg ratio to AMI risk were similar in all age groups (Tables 2 and 3).

A 1 mg/L increment in Mg concentration in groundwater decreased the AMI risk by 4.9% (Table 4), whereas a one-unit increment in Ca:Mg ratio increased the AMI risk by 3.1% (Table 5). Ca and Cr did not have any additional effect on the spatial variation of the AMI incidence.

## Discussion

Our result of the high Ca:Mg ratio in local groundwater associated with an increasing risk of AMI is compatible with previously presented assumptions about a protective role of hard water against CVD (Sauvant and Pepin 2002). A positive correlation between CHD and the estimated Ca:Mg ratio of the diet has also been suggested in several countries (Karppanen et al. 1978). The results of the present study support previous findings of the inverse association of Mg with the AMI risk (Luoma et al. 1983; Punsar and Karvonen 1979; Rubenowitz et al. 2000; Rylander et al. 1991).

Because Ca is usually present in larger amounts, more attention has been paid to the possibility that Ca would be a protective water factor in the etiology of CVD (Karppanen 1981). In Finland, CVD mortality is exceptionally high, and the intake of Ca, 1,187 mg/day (Männistö et al. 2002), is higher than in most countries (Varo 1974). The main sources of Ca intake are milk products (Männistö et al. 2002). Hence, the Finnish situation does not support the hypothesis of a CVD-protective role of Ca. Significantly higher Ca concentrations were noted in the atherosclerotic plagues of abdominal aortas of patients who died of ischemic heart disease than in controls (Vlad et al. 2000). Mg has been shown to be a protective agent against soft tissue calcification, and its protective role in AMI has been well documented (Bloom and Peric-Golia 1989; Vlad et al. 2000).

The underlying mechanisms explaining the effect of Ca and Mg on myosin ATPase are different. Mg is essential to maintain the conformation of enzymatic activity of myosin in cardiac muscle contraction. Ca has a role conducting signals and regulating functions (Saris et al. 2000; Zhu et al. 2002). Mg deficiency may decrease ATPase activity, leading to increases in intracellular Ca and vasoconstriction in the cardiovascular system (Itokawa 2005). Mg is closely involved in maintaining cellular ionic balance through its association with Ca, sodium, and potassium. Mg may influence the binding of other cations, such as Ca, that may have antagonistic or synergistic effects, depending on their concentrations (Saris et al. 2000). Mg deficiency may thus precipitate the development of atherosclerosis and the induction of thrombocyte aggregation (Saris et al. 2000). Residents of soft-water areas have been shown to have lower concentrations of Mg in heart muscle and coronary arteries than do residents of hard-water areas (Anderson et al. 1975; Crawford et al. 1968; Marx and Neutra 1997). This suggests that the content of Mg in the diet is inadequately low. The findings of a positive correlation between CVD mortality and the estimated Ca:Mg ratio of the diet of various countries thus suggest that a high Ca:Mg ratio in the diet may be harmful (Karppanen et al. 1978). Western diets often have a shortage of Mg, and daily intake of Mg does not reach the current recommended daily allowance in many subjects. Therefore, Mg deficiencies are common in industrialized countries (Ford and Mokdad 2003). The recommended dietary allowance for Mg is 350 mg/day (National Nutrition Council 1998). In Finland, the Mg intake is at the recommended level, being on average 405 mg/day (Männistö et al. 2002). However, persons who live in the soft groundwater area, such as residents in eastern Finland, may be at risk for cardiac disease if their dietary Mg intake is low (Klevay and Milne 2002).

In addition to the absolute level of Mg intake, the relation of Mg to the abundance of certain other nutrients in the drinking water and diet may be important for the long-term maintenance of health. In eastern Finland, men who drank water with lower concentrations of Cr had a higher mortality rate from CHD than men in the western part of the country (Punsar et al. 1975). In the same population, the concentrations of serum cholesterol correlated negatively with concentrations of Cr in drinking water (Punsar et al. 1975). Simonoff (1984) reported that plasma Cr levels in patients with coronary artery disease are much lower than those in normal subjects.

In the present study, the total Cr level in local groundwater did not show a statistically significant effect on the risk and spatial variation of AMI. One possible explanation could be the overall small variation of Cr content in well water in Finland. This result confirms the previous findings that in the Finnish diet drinking water was not an important source for Cr supply (Punsar et al. 1977). The estimated safe and adequate daily dietary intake for Cr is 50–200 μg/day for adults (National Research Council 1989). Kumpulainen (1992) reported that in certain developed countries, including Finland, the dietary Cr intake is 50 μg/day or lower.

This study is a population-based ecologic study in which the exposure data connected to each grid cell were measured indirectly by crude estimates of the trace element content of groundwater. In Finland, households in rural areas quite commonly use private well water. However, in this ecologic study, we were not able to determine the extent to which people were served by a public water supply even if they lived in close proximity to their own well. The ecologic studies describe the association between a set of average variables defined in groups of individuals over geographically defined areas. It is important to note that such studies use aggregate data and describe only the association between disease incidence and the average level of exposure to an environmental risk factor, but not the causative role of the factor. Ecologic studies are most useful for generating and testing hypotheses, but biologic or other mechanisms should be determined in further studies. Therefore, the spatial analysis used in the study is appropriate for testing simultaneously the impact of several factors, and the validity of the method used in this study has been demonstrated previously (Karvonen et al. 2002; Kousa et al. 2004; Moltchanova et al. 2004; Rytkönen et al. 2001).

## Conclusions

The result of this ecologic study suggests that at least part of the geographic difference in risk of AMI in Finnish rural areas is related to the Ca:Mg ratio in the drinking (well) water. The high Ca:Mg ratio of water and thus the deficiency of Mg in diet (Karppanen et al. 1978) and in water significantly increase the risk of AMI. It could be assumed that particularly residents of areas with a soft drinking water but high Ca:Mg ratio of drinking water have increased risk of CHD.

## Figures and Tables

**Table 1:** Geochemical constituents in groundwater in Finland.

Element	Mean	Median	SD	2.5%	97.5%
Mg (mg/L)	2.94	2.61	1.15	1.54	5.93
Ca (mg/L)	13.20	12.23	3.99	7.33	23.09
Cr (μg/L)	0.30	0.27	0.11	0.16	0.59
Ca:Mg ratio	5.29	5.39	1.04	3.28	7.26

**Table 2:** Number of AMI cases, population at risk, AMI incidence (cases per 100,000 person-years), and 95% HDR by age and Mg concentration of well water among men in 1983, 1988, and 1993 (pooled data).

Mg (mg/L)	Age (years)	No. of cases	Population at risk	Incidence/100,000/year	95% HDR^a^
< 2.28 (*n* = 938)^b^	35–39	36	38,745	93	64–124
	40–44	73	35,491	206	159–253
	45–49	128	33,212	385	320–453
	50–54	219	31,916	686	596–778
	55–59	293	33,104	885	785–987
	60–64	380	32,767	1,160	1,044–1,277
	65–69	369	29,614	1,246	1,120–1,374
	70–74	373	25,463	1,465	1,317–1,615
	Age standardized	1,871	260,312	622^c^	591–649
2.28–3.12 (*n* = 937)^b^	35–39	70	105,405	66	51–82
	40–44	174	95,994	181	155–208
	45–49	310	85,030	365	324–406
	50–54	460	76,800	599	545–654
	55–59	677	79,417	852	789–917
	60–64	747	80,813	924	859–991
	65–69	845	75,137	1,125	1,049–1,201
	70–74	898	66,501	1,350	1,263–1,439
	Age standardized	4,181	665,097	551^c^	534–568
> 3.12 (*n* = 939)^b^	35–39	147	271,609	54	46–63
	40–44	346	251,959	137	123–152
	45–49	590	220,012	268	247–290
	50–54	938	189,967	494	462–526
	55–59	1,293	188,670	685	648–723
	60–64	1,520	186,976	813	772–854
	65–69	1,788	173,410	1,031	983–1,079
	70–74	1,821	153,480	1,186	1,132–1,241
	Age standardized	8,443	1,636,083	463^c^	454–474

a The 95% posterior HDRs were calculated assuming Poisson distribution of the cases and a vague gamma (0.001, 0.001) prior.

b *n* = number of grid cells.

c Standardized to world standard population.

**Table 3:** Number of AMI cases, population at risk, AMI incidence (cases per 100,000 person-years), and 95% HDR by age and Ca:Mg ratio of well water among men in 1983, 1988, and 1993 (pooled data).

Ca:Mg ratio	Age (years)	No. of cases	Population at risk	Incidence/100,000/year	95% HDR^a^
< 4.90(*n* = 937)^b^	35–39	133	253,701	52	44–61
	40–44	330	235,527	140	125–155
	45–49	562	206,586	272	250–295
	50–54	878	179,436	489	457–522
	55–59	1,222	179,627	680	642–719
	60–64	1,465	178,526	821	779–863
	65–69	1,684	165,988	1,015	966–1,063
	70–74	1,775	146,817	1,209	1,153–1,265
	Age standardized	8,049	1,546,208	464^c^	453–474
4.90–5.73(*n* = 939)^b^	35–39	63	85,478	74	56–92
	40–44	140	78,312	179	150–209
	45–49	231	69,471	333	290–376
	50–54	362	62,428	580	521–640
	55–59	528	63,580	830	760–901
	60–64	589	64,545	913	839–986
	65–69	692	59,639	1,160	1,075–1,248
	70–74	681	52,721	1,292	1,196–1,390
	Age standardized	3,286	536,174	540^c^	521–559
> 5.73(*n* = 938)^b^	35–39	57	76,580	74	56–94
	40–44	123	69,605	177	146–208
	45–49	235	62,197	378	330–426
	50–54	377	56,819	664	597–731
	55–59	513	57,984	885	809–962
	60–64	593	57,485	1,032	949–1,115
	65–69	626	52,534	1,192	1,098–1,285
	70–74	636	45,906	1,385	1,278–1,493
	Age standardized	3,160	479,110	586^c^	564–606

a The 95% posterior HDRs were calculated assuming Poisson distribution of the cases and a vague gamma(0.001,0.001) prior.

b *n* = number of grid cells.

c Standardized to world standard population.

**Table 4:** The estimated effects of Mg, Ca, and Cr on the incidence of the first AMI among Finnish men in 1983, 1988, and 1993 (pooled data).

Element	Posterior mean (%)	95% HDR
Mg (mg/L)	−4.9*	−8.8 to −0.9
Ca (mg/L)	0.9	−0.1 to 2.1
Cr (μg/L)	−10.6	−40.6 to 23.3

The effects were estimated simultaneously in a single spatial model. A 1 mg/L increment of Mg decreases AMI risk by 4.9% when the effects of Ca and Cr are controlled.

* 95% HDR does not include zero.

**Table 5:** The estimated separate effects of Ca:Mg ratio, Mg, Ca, and Cr on the incidence of the first AMI among Finnish men in 1983, 1988, and 1993 (pooled data).

Element	Posterior mean (%)	95% HDR
Ca:Mg ratio	3.1*	0.5 to 5.7
Mg (mg/L)	−3.0	−5.9 to 0.1
Ca (mg/L)	−0.1	−1.0 to 0.8
Cr (μg/L)	−13.2	−46.9 to 16.1

Each effect was estimated in a separate spatial model. One unit increment in Ca:Mg ratio increases AMI risk by 3.1%.

* 95% HDR does not include zero.

## Appendix 1. A Short Description of the Interpolation

### Observations.

Ca, Mg, and Cr concentrations in the Finnish groundwaters (wells and springs; *n* ≈ 4,300 for each element) were obtained from the hydrogeochemical database of the Geological Survey of Finland. For Ca and Mg, only a few samples were below the detection limits, whereas about half of the Cr samples were below the detection limit. The observations could then be divided into two classes: *a*) samples that were above the detection limit (valid) and *b*) samples that were below the detection limit (low). The models allowed us to account for individual and spatial uncertainties of the observations.

In what follows, normal and log-normal distributions are parameterized by precision (τ = inverse variance). The Bayesian hierarchical models were simulated with WinBUGS using a total of 20,000 iterations of which 10,000 were discarded as burn-in. Convergence was assessed visually from the sample paths. The observations were interpolated into 10 × 10 km grid cells across Finland.

### Observations below detection limits.

The low observations were known to lie in the right half-open interval (0, detection limit), leading basically to a uniform error probability distribution for each low observation.

### Valid observations.

The joint errors (of analysis and sampling) for valid observations have been estimated to be ɛ= 15% of the observed value (except ɛ= 20% for 0.2 μg/L ≥Cr ≥1 μg/L), assuming normal distribution and 95% confidence. Using Cr as an example, the precision for each observation was then

giving the measurement error probability distribution for each valid observation as

### Observations in each grid cell.

For a single grid cell, each observation (Ca, Mg, or Cr, both valid and low) was considered log-normally distributed with mean μ*_i_* and a common precision across all cells τ; that is, for Cr:

and

where, in addition to μ*_i_*, the individual error terms (described above) were included in μ^ɛ^*_i, j_*_valid_ and μ^ɛ^*_i, j_*_low_

### The spatial CAR interpolation model.

The interpolated concentration in ground-water was modeled using linear regression:

where α_0_ is the logarithm of the baseline concentration and λ*_i_* has a CAR prior:

where  is the average λ*_i_* over the neighbors of a cell (having *m**_i_* neighbors) and τ_CAR_ is the overall spatial precision. The neighbors were defined as the cells adjacent to a grid cell *i* through side or corner.

An improper flat prior was used for the baseline [*p*(α_0_) ∞ 1] and vague gamma priors were used for the precisions: τ_CAR_ ~ Γ(0.01, 0.01) and τ ~ Γ (0.01, 0.01).

### List of variables

**Table:**

α_0_	Baseline concentration
Ca	Calcium concentration (samples)
Mg	Magnesium concentration (samples)
Cr	Chromium concentration (samples)
ɛ	Joint error of analysis and sampling for valid observations
*i*	Index for grid cells
*j*low	Index for low observations (in a given grid cell)
*j*valid	Index for valid observations (in a given grid cell)
λ*_i_*	The local spatial random effect
*m**_i_*	Number of neighbors for a grid cell *i*
μ*_i_*	Mean of all observations in a grid cell *i*; interpolated concentration ≡ exp(μ*_i_*)
μ^ɛ^*_i_*_,_*_j_*_valid_	Mean of all observations in a grid cell *i* including measurement error term for a valid observation *j*valid
μ^ɛ^*_i_*_,_*_j_*_low_	Mean of all observations in a grid cell *i* including error probability distribution for a low observation *j*low
τ	Common precision for the log-normal distributions in each grid cell
τ_CAR_	Overall spatial precision
τ*_i_*_,_*_j_*_valid_	Measurement error precisions of valid observations

“Observations” is defined here as Ca, Mg, or Cr.

## Appendix 2. The Spatial Disease Model

The model is a full conditional autoregressive model (CAR) with covariates (Kousa et al. 2004). Poisson distributions were assumed for the case counts in each grid cell *i* and age group *k*:

Only inhabited grid cells were taken into account when calculating the Poisson likelihoods. For this study, the grid cells classified as urban were considered uninhabited; that is, there was no risk population.

The Poisson rates (μ*_ik_*) were modeled using a log-linear regression:

where α_0_ is the baseline risk, λ*_i_* is the local spatial random effect, β*_k_* are the age-group–specific risks (nonproportional hazards and β_1_ ≡ 0), ξ is the vector of the geochemical covariate effects, *Z**_i_* is the vector of interpolated geochemical constituents and *N**_ik_* is the risk population. As in the interpolation model (Appendix 1, Equations A1.5 and A1.6 ), the λ*_i_* were assigned a CAR prior:

where  is the average λ*_i_* over the neighbors of a cell (having *m**_i_* neighbors) and τ_CAR_ is the overall spatial precision. The neighbors were defined as the cells adjacent to a grid cell *i* through side or corner.

An improper flat prior was used for the baseline: *p*(α_0_) ∞ 1, and a vague gamma prior for the spatial precision: τ_CAR_ ~Γ (0.01, 0.01).

### List of variables

**Table:**

α_0_	Baseline risk
*i*	Index for grid cells
*k*	Age group
λ*_i_*	The local spatial random effect for grid cell *i*
*m**_i_*	Number of neighbors for grid cell *i*
μ*_ik_*	Poisson rate for grid cell *i* and age group *k*
*N**_ik_*	Risk population for grid cell *i* and age group *k*
τ_CAR_	Spatial precision
ξ	Vector of geochemical covariate effects
*y**_ik_*	Number of cases for grid cell *i* and age group *k*
*Z**_i_*	Vector of geochemical covariates for grid cell *i*
